# PLD2 deletion ameliorates sepsis-induced cardiomyopathy by suppressing cardiomyocyte pyroptosis via the NLRP3/caspase 1/GSDMD pathway

**DOI:** 10.1007/s00011-024-01881-w

**Published:** 2024-04-17

**Authors:** Jun Li, Da Teng, Wenjuan Jia, Lei Gong, Haibin Dong, Chunxiao Wang, Lihui Zhang, Bowen Xu, Wenlong Wang, Lin Zhong, Jianxun Wang, Jun Yang

**Affiliations:** 1 School of Basic Medical Sciences, Qingdao University, No. 308 Ningxia Road, Qingdao, 266071 Shandong China; 2 Department of Cardiology, Yantai Yuhuangding Hospital, Qingdao University, No. 20 Yudong Road, Yantai, 264000 Shandong China

**Keywords:** GSDMD, NLRP3, Phospholipase D2, Pyroptosis, Sepsis-induced cardiomyopathy, Caspase 1

## Abstract

**Objective:**

Sepsis-induced cardiomyopathy (SICM) is a life-threatening complication. Phospholipase D2 (PLD2) is crucial in mediating inflammatory reactions and is associated with the prognosis of patients with sepsis. Whether PLD2 is involved in the pathophysiology of SICM remains unknown. This study aimed to investigate the effect of PLD2 knockout on SICM and to explore potential mechanisms.

**Methods:**

The SICM model was established using cecal ligation and puncture in wild-type and PLD2-knockout mice and lipopolysaccharide (LPS)-induced H9C2 cardiomyocytes. Transfection with PLD2-shRNA lentivirus and a PLD2 overexpression plasmid were used to interfere with PLD2 expression in H9C2 cells. Cardiac pathological alterations, cardiac function, markers of myocardial injury, and inflammatory factors were used to evaluate the SICM model. The expression of pyroptosis-related proteins (NLRP3, cleaved caspase 1, and GSDMD-N) was assessed using western blotting, immunofluorescence, and immunohistochemistry.

**Results:**

SICM mice had myocardial tissue damage, increased inflammatory response, and impaired heart function, accompanied by elevated PLD2 expression. PLD2 deletion improved cardiac histological changes, mitigated cTNI production, and enhanced the survival of the SICM mice. Compared with controls, PLD2-knockdown H9C2 exhibits a decrease in inflammatory markers and lactate dehydrogenase production, and scanning electron microscopy results suggest that pyroptosis may be involved. The overexpression of PLD2 increased the expression of NLRP3 in cardiomyocytes. In addition, PLD2 deletion decreased the expression of pyroptosis-related proteins in SICM mice and LPS-induced H9C2 cells.

**Conclusion:**

PLD2 deletion is involved in SICM pathogenesis and is associated with the inhibition of the myocardial inflammatory response and pyroptosis through the NLRP3/caspase 1/GSDMD pathway.

**Supplementary Information:**

The online version contains supplementary material available at 10.1007/s00011-024-01881-w.

## Introduction

Despite improvements in critical care, sepsis affects more than 50 million individuals and accounts for 11 million deaths annually, making it a leading causes of death worldwide [1, 2]. Most patients with sepsis have abnormal cardiac function, frequently referred to as “sepsis-induced cardiomyopathy” (“SICM”) [3, 4]. SICM is more prevalent, with an incidence rate ranging from 30 to 50%, and has a two- to three-fold higher mortality rate than that of sepsis without cardiac injury [4, 5]. SICM is defined as a decreased ejection fraction, but is reversible [6, 7]. Patients with SICM may survive with prompt and efficient therapy. Its pathophysiology is complicated and involves numerous mechanisms, one of which is the myocardial inflammatory response, which results in cardiomyocyte abnormalities [8, 9]. However, the mechanisms underlying inflammation are poorly understood. Therefore, an in-depth understanding of the pathogenesis of inflammation is essential to improve the prognosis of patients with SICM.

Phospholipase D2 (PLD2) is a lipid hydrolase widely found in mammals [10]. It is a key intracellular signaling effector protein required for cellular resistance to microbial invasion and inflammatory responses [11, 12]. It primarily localizes to sarcolemmal membranes [13] and is involved in the development of numerous cardiac disorders such as ischemia–reperfusion damage [14], congestive heart failure [15], and cardiac hypertrophy [16]. However, the role of PLD2 in SICM has not been reported. Our previous studies demonstrated that PLD2 knockout improved the survival of mice with sepsis [17]. SICM is closely linked to sepsis prognosis. Consequently, there may be a connection between the protective effect of PLD2 knockdown and a possible improvement in SICM. The inflammatory response is important in the pathogenesis of SICM, and PLD2 is associated with inflammation [18]. Hence, the theory that PLD2 knockout improves the myocardial inflammatory response in SICM and thereby increases survival is feasible. However, this issue requires further confirmation.

Pyroptosis is a form of programmed cell death characterized by its pro-inflammatory nature, which sets it apart from other forms of cell death, such as apoptosis and necroptosis [19, 20]. In addition to cell death, pyroptosis results in severe inflammatory damage [21, 22]. Activation of the nod-like receptor family pyrin domain-containing 3 (NLRP3) inflammasome results in the conversion of pro-caspase 1 into cleaved caspase 1 [23]. The inflammasome subsequently cleaves the gasdermin family of proteins (e.g., gasdermin D [GSDMD]) into GSDMD-N, ultimately leading to pyroptosis [24]. The inflammatory response triggered by pyroptosis contributes significantly to myocardial injury in SICM [25]. Multiple mechanisms involved in cardiomyocyte pyroptosis and PLD2 are strongly associated with inflammatory responses. However, whether PLD2 improves cardiac function in mice through lessening cardiomyocyte pyroptosis remains unknown.

We hypothesized that PLD2 deletion improves the prognosis of SICM by decreasing the inflammatory response and cardiomyocyte damage and that pyroptosis may play a role in this process. Experiments on animals and cells have been performed to test this hypothesis.

## Materials and methods

### Mice and grouping

Wild type (WT) and PLD2^−/−^ mice (6–8 weeks) were purchased from Gempharmatech Co., Ltd. (Jiangsu, China). The heterozygous (PLD2^±^) mice obtained from mating WT and PLD2^−/−^ mice were further bred to obtain the mice required for this experiment. Male WT and PLD2^−/−^ offsprings (6–8 weeks) from the same litter were used as the control and experimental groups, respectively. The animal protocols were approved by the Institutional Animal Care and Use Committee of Yantai Yuhuangding Hospital.

For the purpose of observing the changes in myocardial damage and PLD2 expression, WT mice were randomly assigned to the control and cecal ligation and puncture (CLP) groups (n = 6 per group).

### Animal model

Septic cardiomyopathy was generated in mice using a cecal ligation and puncture (CLP) model, following the procedures described in a prior study [26, 27]. In brief, mice were put through a small animal anesthesia machine (Shenzhen Ruiwode Lift Technology Co.,Ltd. China, R5301E), which inhaled 2% isoflurane to induce anesthesia before the procedure and maintained the induction of anesthesia during the procedure. The cecum was fully exposed after a 1 cm longitudinal incision in the lower abdomen of the mice. Two punctures were created using a 22-gauge needle at the site of cecum ligation to induce SICM. A 4–0 suture was used for suturing the distal three-fourths of the cecum. After the stool was extruded, the cecum was patched and sutured with 4–0 silk. The mice were administered 1 ml of 37 °C normal saline solution immediately after surgery and put on a 37 °C thermostatic plate for rewarming until the anesthetic wore off. The mortality of mice was to be monitored during the subsequent investigation.

### Cell culture and treatment

H9C2 was purchased from ATCC (Manassas, VA, USA), and cells were cultured in Dulbecco's modified Eagle's medium supplemented with 10% fetal bovine serum (FBS) at a temperature of 37 °C in a 5% CO_2_ environment. H9C2 cells were treated with LPS (Aldrich Biotechnology Ltd, USA, L2880, *Escherichia coli* O55:B5) at a concentration of 10 μg/ml for 12 h to induce cardiomyocyte damage. Cells were expanded to a density of around 70% before being harvested in order to evaluate the effect of PLD2 knockdown on LPS-induced cardiomyocyte injury.

### Pathological analysis

The mice heart tissue Sects. (5 μm) were deparaffinized and dehydrated, and hematoxylin and eosin (HE) staining was performed according to the manufacturer's protocol (Beijing Solarbio Science & Technology Co., Ltd, China, G1120).

### Echocardiography

Approximately 24 and 48 h after CLP, cardiac functions were evaluated utilizing the Doppler echocardiography (VINNO 6 LAB, VINNO, China). Initially, anesthesia was induced in the mice using a tiny animal anesthetic machine with a 2% concentration of induction isoflurane. This allowed the animals to remain unconscious and immobile state throughout the ultrasonography scan. Heating platforms were used to maintain thermal comfort for the mice. The M-mode tracings were obtained using the transthoracic 2D M-mode echocardiographic equipment. Data had been recorded for heart rate, left ventricular internal dimensions at end-diastole (LVIDd), left ventricular internal dimensions at systole (LVIDs), internal volume at diastole (LVEDV), and internal volume at systole (LVESV). The left ventricular fraction shortening (LVFS) and ejection fraction (LVEF) were subjected to analysis sequentially.

### Cell viability

The effects of LPS on cell viability were determined by the Cell Counting Kit-8 (CCK8) assay. H9C2 cells were seeded into 96-well plates and stimulated with varying doses of LPS (0.5, 1, 2.5, 5, 7.5 10, 15, 20 μg/ml) respectively. Cells were incubated for further 12 h, then CCK8 assay were performed according to the manufacturer's instructions (Nanjing Vazyme Biotech Co., Ltd, China). Cell viability was calculated using the following formula: percentage of cell viability = (A_treatment group−_A_blank group_)/(A_control group−_A_blank group_) × 100%. Each group was evaluated using six replicate wells.

### Immunohistochemistry assay

The heart tissue Sects. (5 µm) were deparaffinized and dehydrated. Subsequently, they were treated by 3% H_2_O_2_ and citrate buffer to block endogenous peroxidase activity and repair the antigen, respectively. Then the tissue was incubated with goat serum for an hour at room temperature, followed by incubating with primary antibodies against NLRP3 (ImmunoWay Biotechnology Company, USA, YT5382) at 4 °C overnight. Finally, the tissue were incubated with secondary antibody (HRP-labeled goat anti-rabbit and anti-mouse IgG mixture) and developed using DAB solution for color visualization. Stained tissue were visualized on microscope and protein expression level were evaluated with Image J.

### Immunofluorescence

Deparaffinized, dehydrated, and antigenic thermal repair with a citric acid solution was applied to mouse heart tissue slices. After being sealed with goat serum for an hour, the pieces were naturally cooled to room temperature. The initial antibody cleaved caspase 1 (Proteintech Group, Inc., 22915-1-AP, 1:200) and GSDMD-N (Bioss, China, bs-14287R, 1:200) were incubated at 4 °C for 12 h. After PBS-washing the primary antibody, it is incubated for an hour in the dark with a fluorescent secondary antibody (Cell Signaling Technology, USA, 8889S or 4412S, 1:200). Before microscopic inspection and photography, slices were blocked with a fluorescence quencher, and DAPI was applied dropwise.

Under sterile conditions, cellular immunofluorescence was performed by placing sterilized round glass coverslips in the 24-well cell culture plates, seeding H9C2 cells on the round glass coverslips, allowing them to grow to 70%, harvesting the cells, and fixing them with 4% paraformaldehyde. The primary antibodies NLPR3 (Boster Biological Technology Co., ltd, China, BA3677, 1:200), cleaved caspase 1, and GSDMD-N were then incubated overnight at 4 °C. Subsequently, the cells were stained with DAPI and goat anti-rabbit Alexa Fluor antibody. Finally, pictures were captured using a fluorescence microscope (Olympus, Japan).

### Western blotting analysis

Cell lysis was collected and protein concentrations were calculated using a BCA assay kitt (Solarbio, China, P0010S). Equal amounts of proteins were subjected to SDS-PAGE and separated at 80 V for 30 min and 110 V for 100 min, then transferred to 0.22 μm nitrocellulose membranes and subsequently probed with the following primary antibodies: PLD2 (ImmunoWay Biotechnology Company, USA, YT3620, 1:1000), NLRP3(1:1000), cleaved caspase 1(1:1000); GAPDH (Sangon Biotech, China, D110016, 1:6000). All experiments were independently replicated three times.

### Quantitative real-time PCR

Total RNA was extracted from cultured cells or mouse heart tissues using TRIzol reagent, and 1 μg of total RNA was reverse-transcribed from RNA to cDNA using the Hifair III First Strand cDNA System (Yeasen Biotechnology Co., Ltd, China, 11141ES60). The relative expression data were gathered using the 2^−△△Ct^ technique. Each experiment was carried out three times with biological replicates. In Table 1, the primers are illustrated.

**Table 1:** Primers Sequences for RT-PCR

Gene	Forwards primer (5′ to 3′)	Reverse primer (5′ to 3′)
IL-6 (mouse)	GCCTTCTTGGGACTGATGCT	GACAGGTCTGTTGGGAGTGG
IL-1β (mouse)	GCTTCAGGCAGGCAGTATCA	AAGGTCCACGGGAAAGACAC
IL-1β (rat)	AGCTGTGGCAGCTACCTATG	GGTCGTCATCATCCCACGAG
TNF-α (mouse)	GATCGGTCCCCAAAGGGATG	CCACTTGGTGGTTTGTGAGTG
TNF-α (rat)	GATCGGTCCCAACAAGGAGG	GCTTGGTGGTTTGCTACGAC
IL-18 (rat)	CAAAAGAAACCCGCCTGTGT	ATAGGGTCACAGCCAGTCCT
GAPDH (mouse)	TGATGGGTGTGAACCACGAG	GCCCTTCCACAATGCCAAAG
GAPDH (rat)	GCGAGATCCCGCTAACATCA	CTCGTGGTTCACACCCATCA

### Evaluation of inflammatory cytokines and assay for lactate dehydrogenase release

After mice were anesthetized, blood from the ocular venous plexus was collected and centrifuged at 1500 g for 20 min, and the serum was separated into fractions for storage and utilization. The ELISA kits (Shanghai Enzyme-linked Biotechnology Co., Ltd, Shanghai, China, ml002095; ml098416; CK-E20324) were used as directed by the manufacturer. To detect cardiac cytotoxicity, lactate dehydrogenase (LDH) in the H9C2 cell supernatant and mouse serum was detected utilizing the LDH cytotoxicity assay reagent (Jiancheng, Nanjing, China, A020-2) according the manufacturer's protocol.

### RNA interference

Small hairpin (sh)RNA targeting rat PLD2 (shPLD2) and a negative control shRNA (shNC) were cloned into miRZip^™^ shRNA Expression Lentivector (Systembio, Shanghai, China). The target sequence of rat PLD2 was 5'- CCGCCTCCTGACCATGTC-3'. The lentivirus particles were generated by HEK293T cells. Following a 48–72 h incubation period in the presence of lentivirus, a concentration of 2 µg/mL puromycin was introduced into H9C2 cells to facilitate the identification of cells exhibiting positive characteristics.

### Overexpression of PLD2

The full length of rat PLD2 was inserted in p3XFLAG-CMV-PLD2 vector and the plasmids were transfected into H9C2 cells using the transfection agent Lipofectamine 3000.

### Electron microscopy

To facilitate the observation of cardiomyocyte pyroptosis using a scanning electron microscope (SEM), the H9C2 cardiomyocytes were challenged to 12 h stimulation with lipopolysaccharide (LPS). In brief, the cells were subjected to fixation in a 2.5% glutaraldehyde solution for 72 h. The cells were then washed and fixed in a 1% osmic acid solution at 4 ℃ for 2 h. The cells were finally imaged using a HITACHI Regulus 8100 SEM. Following drying using a critical point drier and applying a coating using an ion sputtering device, the specimens were subsequently positioned on the stage of an SEM, transported into a designated sample chamber, and captured using an HITACHI Regulus 8100 SEM.

### Statistical analysis

Statistical analysis was carried out by GraphPad Prism 9.0 software from San Diego, California. The data were presented as mean ± SEM. Student's t-test was used for comparing two groups. One-way and two-way ANOVA were utilized for comparing multiple groups, and LSD (least significant difference) was used for comparison between groups. Statistical significance was defined as *p* < 0.05 (two-tailed).

## Results

### Increased expression of PLD2 in cardiac tissue was correlated with decreased heart function in SICM mice

**Fig. 1:**
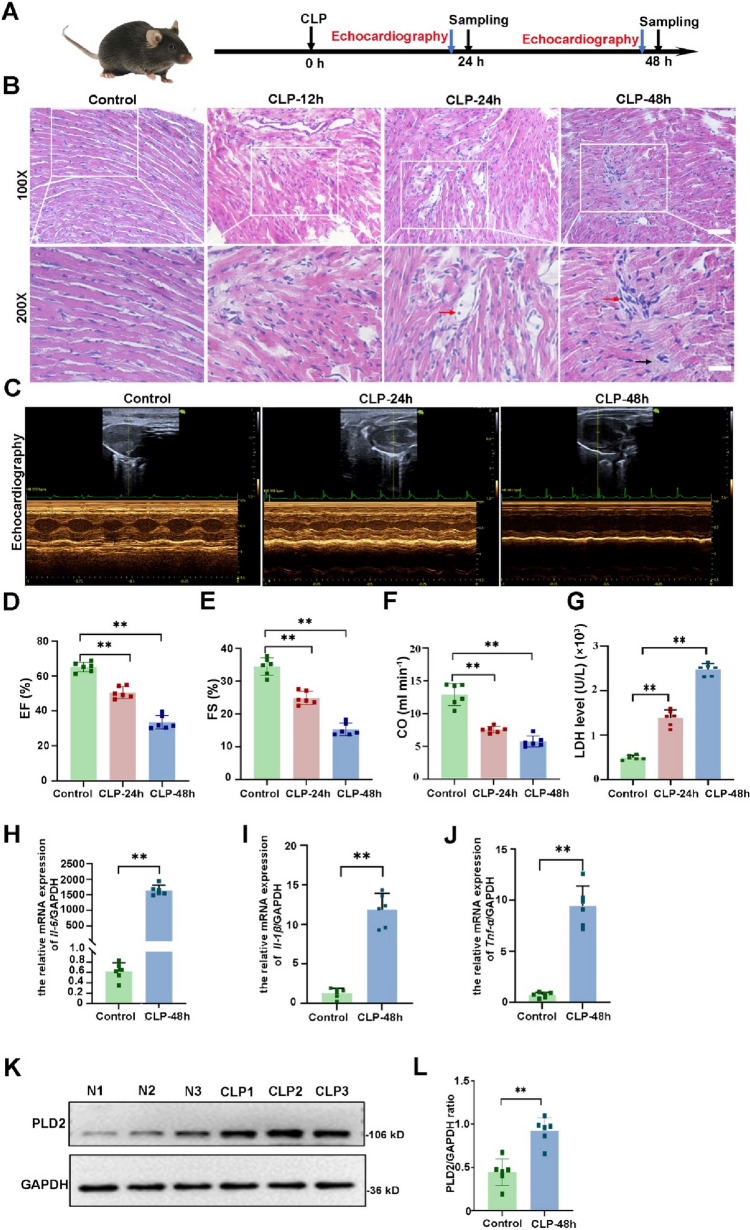
Increased expression of PLD2 in cardiac tissue was correlated with decreased heart function in SICM mice. SICM was induced in mice by utilizing cecal ligation and puncture (CLP). **A** The experimental strategy and timetable for the SICM model are shown in **(A)** (n = 6 mice/group). Histopathological analysis via HE staining of mouse heart section at different points (CLP-12 h, CLP-24 h and CLP-48 h) after CLP is also shown (**B**). CLP induction in SICM mice showed a more disorganized arrangement of cardiomyocytes, along with interstitial edema (black arrows) and some infiltration of inflammatory cells (red arrows). **C** Representative left ventricular echocardiographic M-mode images in mice with control and CLP-induced SICM groups. **D**–**F** Sequential measurements of left ventricular ejection fraction (LVEF), left ventricular shortening fraction (LVFS), and cardiac output (CO) of mice in each group after sham or CLP. **G** Serum levels of lactate dehydrogenase (LDH) in mice among groups. mRNA expression levels of various groups of inflammatory factors, including *Il-6* **(H)**, *Il-1β* **(I)**, and *Tnf-α* **(J),** in mouse cardiac tissue. Western blotting evaluates the expression of PLD2 protein in cardiac tissues of control mice and SICM mice induced by CLP-48 h **(K**, **L)**. Magnification, × 100; scale bar, 100 μm. Magnification, × 200; scale bar, 50 μm. Data represent the mean ± SD of three independent experiments. **P < 0.001 compared to the control group

The SICM model was created by using CLP. The experimental strategy and timetable for the SICM model are shown in Fig. 1A. The examination of the cardiac tissue using HE staining of SICM mice showed a more disorganized arrangement of cardiomyocytes, along with interstitial edema and some infiltration of inflammatory cells. The histological damage was found to be most severe after 48 h compared to that at 12 and 24 h (Fig. 1B). Echocardiographic parameters are shown in Supplementary Table 1. The results revealed that CLP mice had reduced cardiac output (CO), LVFS, and LVEF at 48 h compared to that at 24 h (Fig. 1C–F). Levels of LDH, a marker of myocardial injury, were substantially elevated at 48 h (Fig. 1G). Therefore, the SICM model was selected for the follow-up investigation 48 h after CLP. *Tnf-α, Il-1β*, and *Il-6* inflammatory indicators in cardiac tissues, identified with reverse transcription-polymerase chain reaction (RT-PCR), were substantially greater in the CLP group than in the control group (Fig. 1H–J). This suggested that the SICM model was successfully established.

Furthermore, we detected PLD2 expression in mouse heart tissues. Western blotting results demonstrated that PLD2 expression was considerably elevated in the CLP mice than in the control mice (Fig. 1K, L), indicating that PLD2 may be involved in the initiation and progression of SICM.

### *PLD2 knockdown protected cardiomyocytes from LPS-induced damage and inflammation *in vitro

**Fig. 2:**
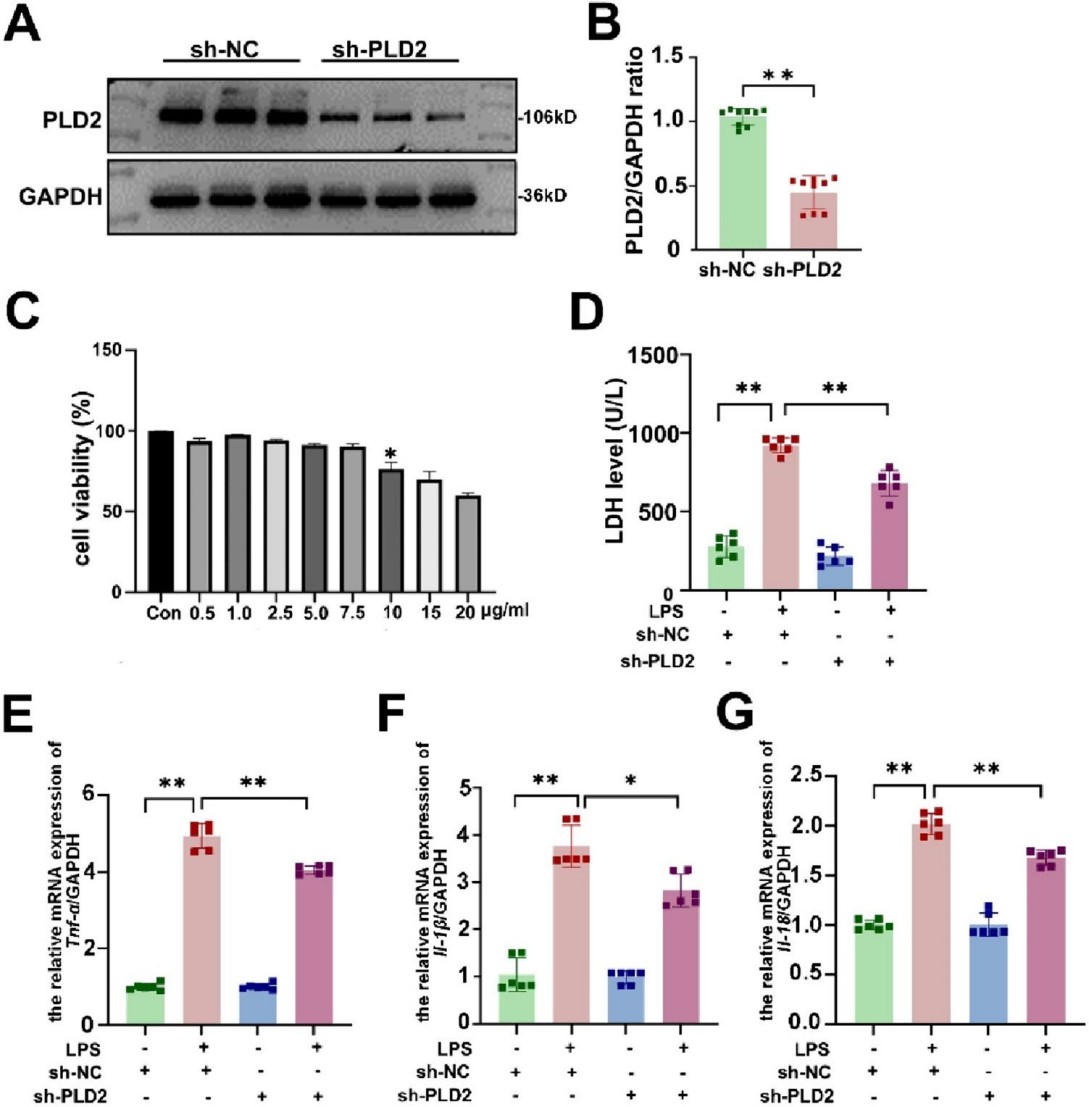
PLD2 knockdown protected cardiomyocytes from LPS-induced cardiac damage and inflammation in vitro. The PLD2 gene was targeted for knockdown using shRNA to establish the H9C2 stable cell line. The H9C2 cells were transfected with control (sh-NC) or PLD2 shRNA (sh-PLD2) for 48 h and then exposed to LPS (10 μg/ml) for 12 h. The cells were divided into four groups: sh-NC, sh-NC + LPS, sh-PLD2, and sh-PLD2 + LPS. The efficiency of the knockdown was assessed using western blotting analysis (**A**, **B**). The impact of varying concentrations of LPS on the viability of H9C2 cardiomyocytes was assessed using the CCK8 assay (**C**). The asterisk indicates that when the concentration of LPS was 10 μg/ml, cell viability dropped dramatically to 76.5%. LDH level in cell supernatants (**D**) and the mRNA expression levels of inflammatory markers *Tnf-α* (**E**), *Il-1β* (**F**), and *Il-18* (**G**) in H9C2 cells. **P < 0.001 compared to the sh-NC group; *P < 0.05, **P < 0.001 compared to the sh-PLD2 + LPS group

The LPS-induced H9C2 cardiomyocytes were used in an in vitro cell-damage model of SICM. As depicted in Fig. 2C, the cytotoxicity of LPS on H9C2 cardiomyocytes at 12 h was dose-dependent, when the concentration of LPS was 10 μg/ml, cell viability dropped dramatically to 76.5%, hence 10 μg/ml was chosen for the following experimental investigation. The following experiments were conducted at this concentration. Initially, using shRNA to knockdown the PLD2 gene, we established an H9C2 stable cell line and confirmed the knockdown efficacy of the cells using western blotting (Fig. 2A, B). The results revealed that the expression of PLD2 was dramatically decreased in the sh-PLD2 group. To further validate the effect of PLD2 knockdown on cardiomyocyte injury, we measured LDH in the supernatant of cardiomyocytes. The results showed that LDH production was significantly lower in the sh-PLD2 + LPS group than in the sh-NC + LPS group (Fig. 2D). In addition, RT-PCR revealed that PLD2 knockdown decreased the production of inflammatory factors *Tnf-α* (Fig. 2E), *Il-1β* (Fig. 2F), and *Il-18* (Fig. 2G).

### *PLD2 deficiency alleviated NLRP3/caspase 1/GSDMD-mediated pyroptosis in cardiomyocytes induced by LPS *in vitro

**Fig. 3:**
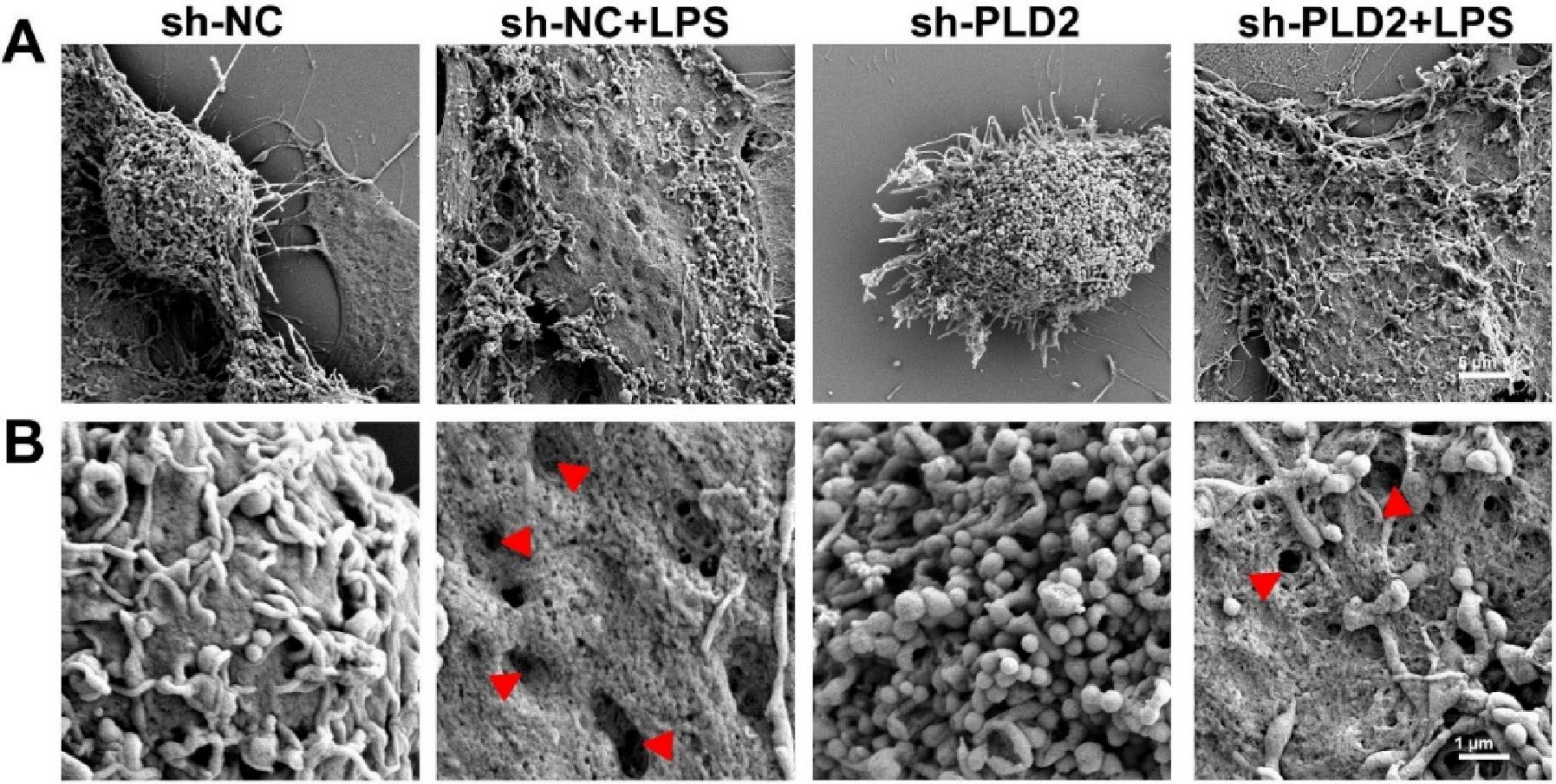
PLD2 deficiency alleviated cardiomyocytes pyroptosis induced by LPS in vitro. Scanning electron micrograph of H9C2 cells after 12 h of treatment with 10 μg/ml LPS. There were four distinct cell groups: sh-NC, sh-NC + LPS, sh-PLD2, and sh-PLD2 + LPS. As shown in Figure **A**, at a magnification of 2000 × , control cells are characterized by a rounded cellular mass with extended cellular processes. The cells treated with LPS exhibit irregular margins and lack processes. Plasma membranes appear ruptured. At a magnification of 10,000 × (**B**), the surface of control cells exhibits projections from the plasma membrane and extracellular vesicles. The stimulation of LPS induces the creation of pits and pores of varying sizes, and a pronounced loss of extracellular vesicles (red arrow). The degree of holes and pits in the membrane was reduced in the sh-PLD2 + LPS group as compared to the sh-NC + LPS group. Scale = 5 μm (**A**) and 1 μm (**B**)

Scanning electron microscopy was used to evaluate the detailed surface morphology and pyroptosis of H9C2 cells (Fig. 3). The control cells showed a distinctive morphology with spherical cells, cellular protrusions, plasma membrane protrusions, and extracellular vesicles on the surface. By contrast, cells treated with LPS showed irregular margins, absence of processes, formation of pits and pores of different sizes, and a significant decrease in extracellular vesicles. Furthermore, deletion of the PLD2 gene led to enhanced membrane pits and holes, leading to alleviate cellular pyroptosis.

**Fig. 4:**
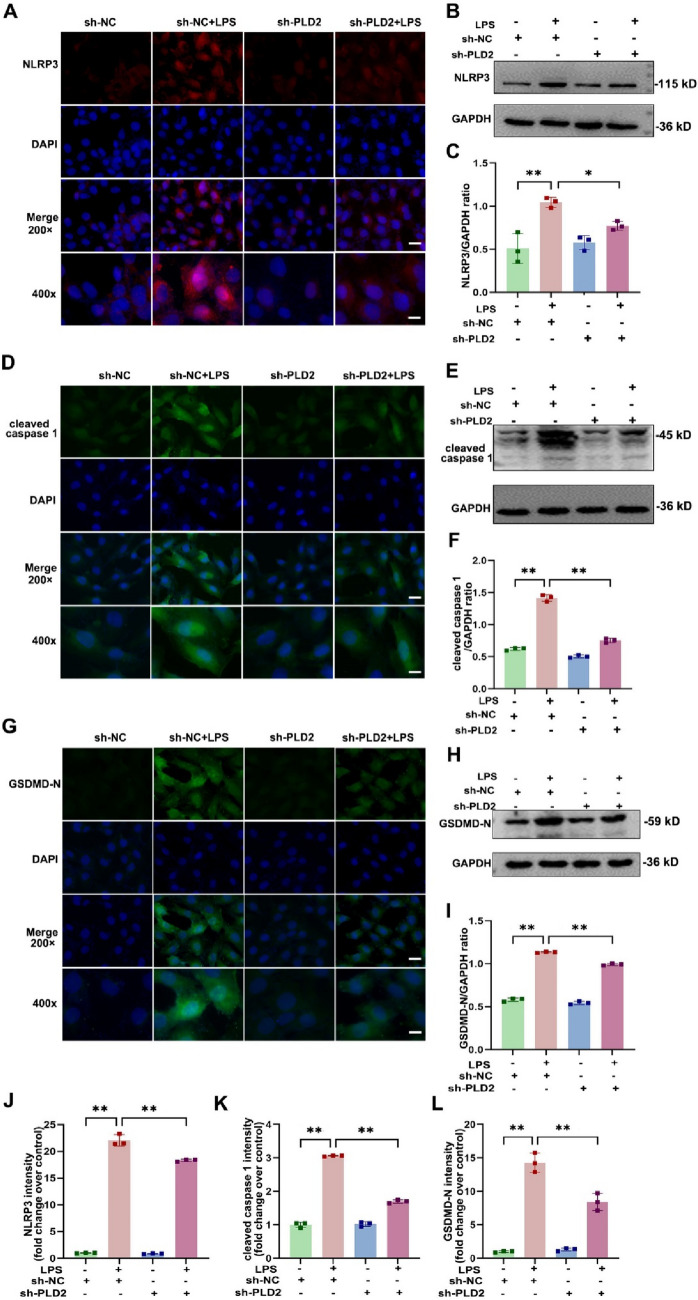
PLD2 deficiency alleviated NLRP3/caspase 1/ GSDMD-mediated pyroptosis in cardiomyocytes induced by LPS in vitro. The H9C2 cells were treated with 10 μg/ml LPS for 12 h and were divided into four distinct groups, namely sh-NC, sh-NC + LPS, sh-PLD2, and sh-PLD2 + LPS. The graphical depiction of the immunofluorescence findings for NLRP3, cleaved caspase 1, and GSDMD-N of the cells in different groups is shown by (**A**, red), (**D**, green), and (**G**, green), respectively. The related bar graphs of fluorescence intensity analysis are represented by **J**, **K**, and **L**, respectively. Western blotting analysis revealed the protein expression levels of NLRP3 (**B**, **C**), cleaved caspase 1 (**E**, **F**), and GSDMD-N (**H**, **I**), in the order mentioned. Magnification, × 200; scale bar, 50 μm. Magnification, × 400; scale bar, 20 μm. **P < 0.001 compared to the sh-NC group; *P < 0.05, **P < 0.001 compared to the sh-PLD2 + LPS group

To further elucidate the specific mechanism of PLD2, we validated the expression of pyroptosis-associated proteins. Cellular immunofluorescence data demonstrated that the sh-PLD2 + LPS group had lower NLRP3 expression in H9C2 than the sh-NC + LPS group (Fig. 4A, J); western blotting results were consistent with the immunofluorescence results (Fig. 4B, C). Caspase-1 and GSDMD are critical downstream molecules of NLRP3, participating in the occurrence of pyroptosis. When PLD2 was silenced, we observed a significant reduction in the expression of cleaved caspase-1 and GSDMD-N (Fig. 4D–I, K, L). These findings indicated that PLD2 knockdown may decrease cardiac pyroptosis in SICM by involving the NLRP3/caspase 1/GSDMD pyroptosis pathway.

### *PLD2 overexpression exacerbates cardiomyocyte injury through NLRP3-mediated inflammatory response *in vitro

**Fig. 5:**
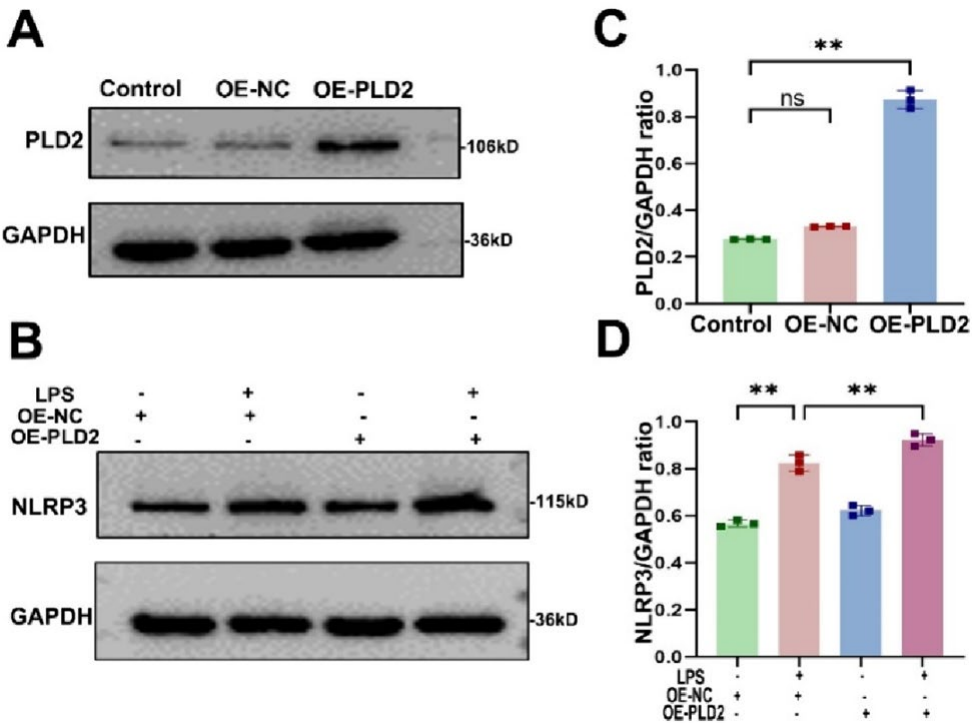
PLD2 overexpression exacerbates cardiomyocyte injury through NLRP3-mediated inflammatory response in vitro. A plasmid vector was used for the purpose of overexpressing PLD2 and confirming the effective completion of the constructed plasmid (**A**, **B**). To investigate the role of PLD2 gene overexpression in LPS-induced myocardial injury, cells were divided into four distinct groups, namely OE-NC, OE-NC + LPS, OE-PLD2, and OE-PLD2 + LPS. Western blotting shows the different groups' levels of NLRP3 expression after 10 μg/ml LPS for 12 h stimulation (**C**, **D**). **P < 0.001 compared to the OE-NC group; **P < 0.001 compared to the OE-NC + LPS group

To further clarify the protective role of PLD2 deletion against LPS-mediated cardiomyocyte damage, we employed plasmid vectors for overexpressing PLD2 and transfected them into H9C2 cells. The western blotting results demonstrated successful overexpression of PLD2 (Fig. 5A–C). Of note, in the context of PLD2 overexpression, there is a concurrent increase in NLRP3 expression within the PLD2-OE + LPS group (Fig. 5B–D). These findings implied that PLD2 overexpression may worsen cardiac damage and inflammatory responses by promoting NLRP3-mediated pyroptosis in cardiomyocytes.

### PLD2 deletion reduced CLP-induced cardiac tissue damage and mortality in SICM mice

**Fig. 6:**
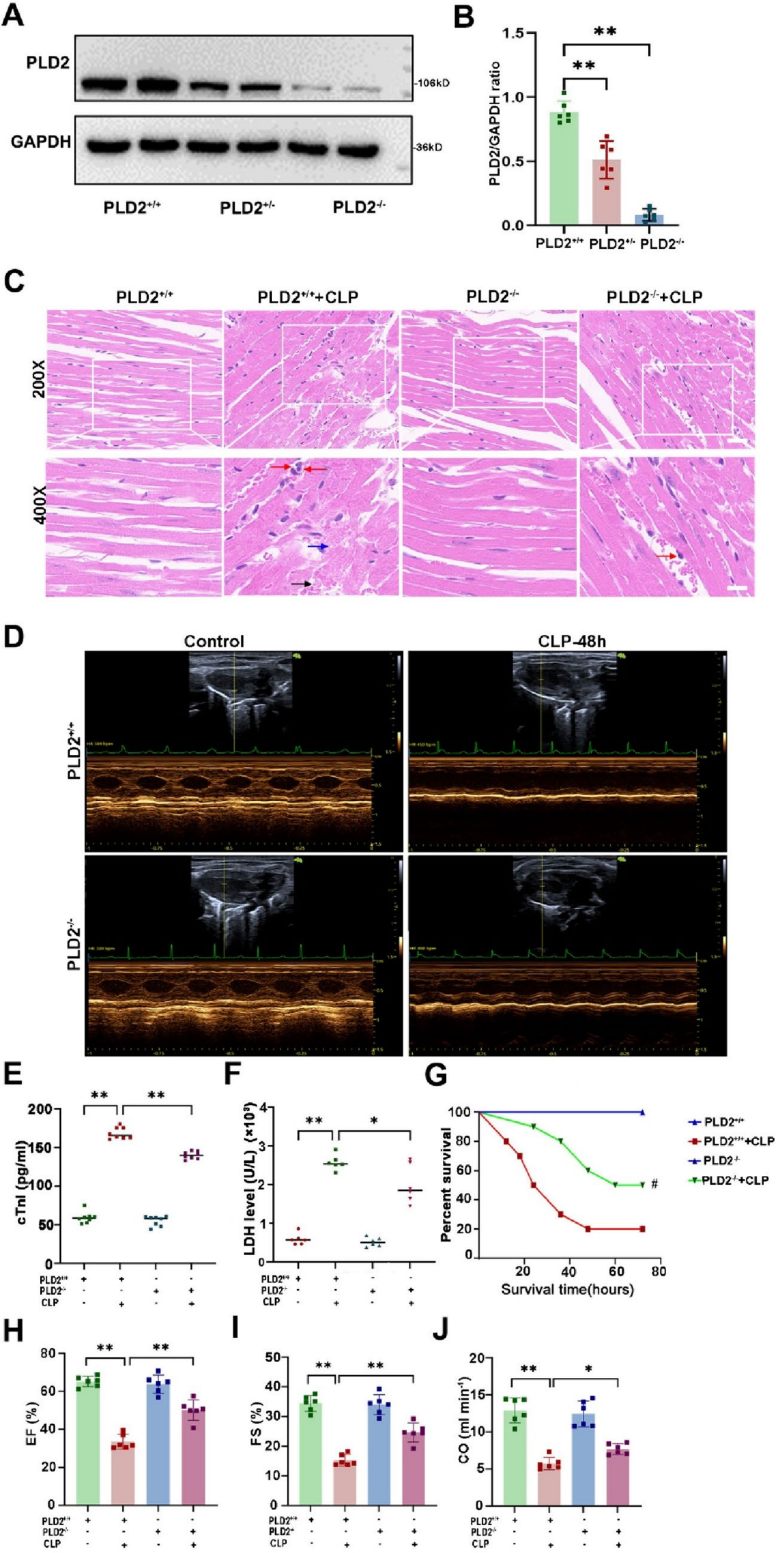
PLD2 deletion reduced CLP-induced cardiac tissue damage and mortality in SICM mice. The SICM model was constructed 48 h after CLP. Efficient creation and validation of PLD2 gene knockout in mouse heart tissue by western blotting (**A, B**). The mice were allocated into four distinct groups, namely PLD2^+/+^, PLD2^+/+^  + CLP, PLD2^−/−^, PLD2^−/−^ + CLP (n = 6 mice/group). Histopathological alterations in the cardiac tissues of four distinct groups of mice were evaluated using the HE staining technique (**C**). CLP-induced SICM caused cardiac tissue edema (blue arrows), inflammatory cell infiltration (red arrows), and blood congestion (black arrows). Cardiac function was evaluated by representative left ventricular echocardiographic M-mode pictures in mice (**D**), specifically by measuring parameters such as LVEF (**H**), LVFS (**I**), and CO (**J**). Changes in the expression level of cTNI, a marker of myocardial injury, in mouse serum by ELISA (**E**) and LDH (**F**) by LDH cytotoxicity assay reagent. The survival of sham mice and LPS-induced SICM mice was recorded over 72 h (**G**). Magnification, × 200; scale bar, 50 μm. Magnification, × 400; scale bar, 20 μm. **P < 0.001 compared to the PLD2^+/+^ group; *P < 0.05, **P < 0.001 compared to the PLD2^+/+^  + CLP group

PLD2 knockout mice were successfully established, as shown in Fig. 6A, B. Initially, we examined the survival rates of WT and PLD2^−/−^ SICM mice. To assess mortality in mice, we constructed a more severe model of SICM in which four-fifths of the distal cecum was ligated during CLP surgery. The mice began to die 12 h after CLP surgery. Forty-eight hours later, PLD2^−/−^ SIMC mice had a higher survival rate than that of the WT mice (46% vs 22%, *p* = 0.0487; Fig. 6G). Compared to PLD2^+/+^ group, PLD2^−/−^ mice exhibited significantly ameliorated pathological changes such as disorganized arrangement of cardiomyocytes, interstitial edema, and inflammatory cell infiltration in myocardial tissue when induced by CLP (Fig. 6C). Echocardiographic parameters are shown in Supplementary Table 2. Compared to PLD2^+/+^ SICM mice, the ejection fraction, fractional shortening, and cardiac function indicators improved in the PLD2^−/−^ SICM mice, based on cardiovascular ultrasonography (Fig. 6D and H–J). Biomarkers of myocardial injury, such as cardiac troponin I (cTnI) (Fig. 6E) and LDH (Fig. 6F) levels, were significantly decreased in the PLD2^−/−^ SICM mice. Based on these data, PLD2 gene-specific deletion seems to reduce heart damage and mortality in SICM mice.

### PLD2 deletion alleviated cardiac tissues inflammatory response and NLRP3/caspase 1/GSDMD-mediated pyroptosis in CLP-induced SICM mice

**Fig. 7:**
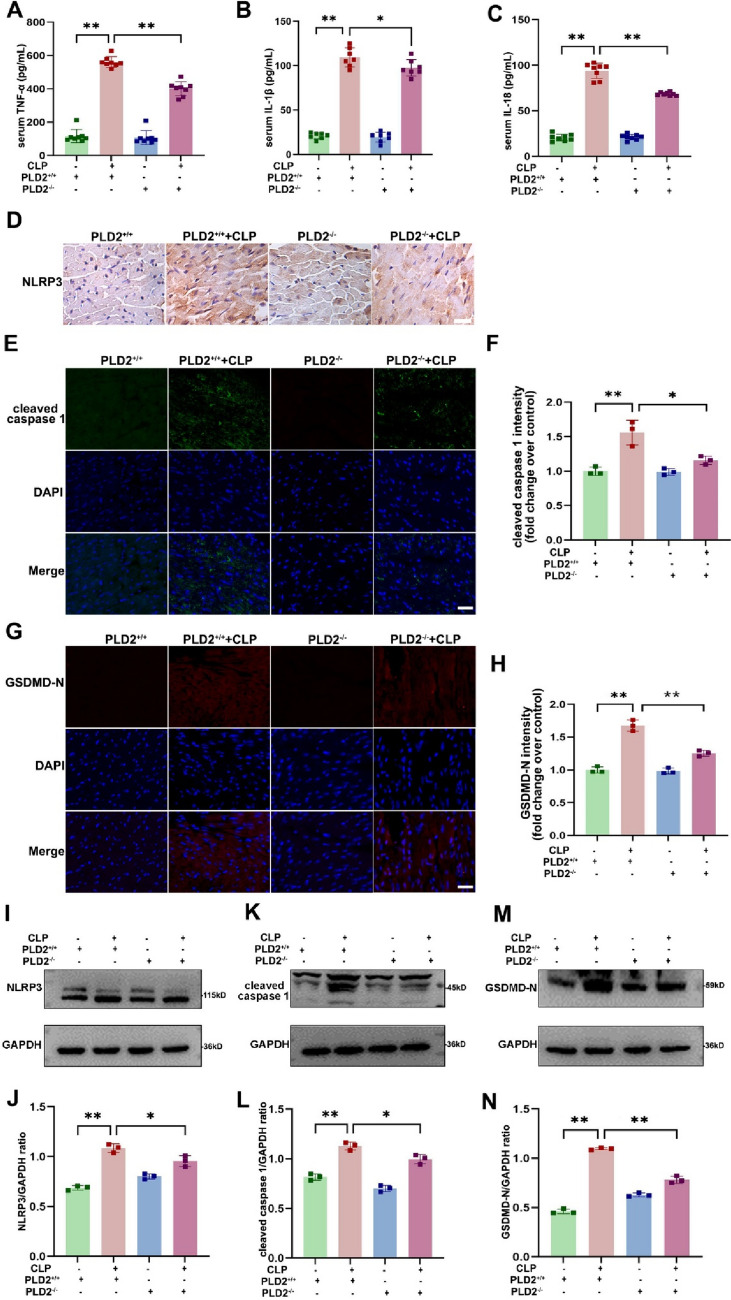
PLD2 deletion alleviated cardiac tissues inflammatory response and NLRP3/caspase 1/GSDMD-mediated pyroptosis in CLP-induced SICM mice. The SICM mice were created 48 h after the CLP. The mice were allocated into four distinct groups, namely PLD2^+/+^, PLD2^+/+^  + CLP, PLD2^−/−^, PLD2^−/−^ + CLP (n = 6 mice/group). The effect of PLD2 deletion on the levels of serum inflammatory factors (*Tnf-α, Il-1β*, and *Il-18*) in SICM mice was investigated using ELISA (**A**–**C**). **D** Immunohistochemistry assay shows NLRP3 distributions in the heart tissue of mice. Distributions of pyroptosis-associated proteins cleaved caspase 1 (**E**, green), GSDMD-N (**G**, red) and associated fluorescence intensity analysis of cleaved caspase 1 (**F**), and and GSDMD-N (**H**) in the heart of mice among the groups. **I**, **K, M** Western blotting analysis to determine NLRP3, cleaved caspase 1, and GSDMD-N levels in mice from each group. Quantifications of NLRP3, cleaved caspase 1, and GSDMD-N are shown in (**J**), (**L**), and (**N**) respectively. Magnification × 200, scale bar, 50 μm. **P < 0.001 compared to the PLD2^+/+^ group; *P < 0.05, **P < 0.001 compared to the PLD2^−/−^ + CLP group

During pyroptosis, cells release inflammatory factors into circulation. To assess the influence of PLD2 deletion on inflammation, we examined the levels of inflammatory mediators TNF-a, IL-1β, and IL-18 in mouse serum by ELISA. We noted reduced levels of these inflammatory markers in the PLD2^−/−^ + CLP group compared to those in the PLD2^+/+^  + CLP group (Fig. 7A–C). We also investigated the expression of proteins associated with pyroptosis. The immunohistochemistry demonstrated that PLD2 deletion reduced the expression of NLRP3 in cardiac tissues of SICM mice induced by CLP, compared to wild-type mice (Fig. 7D). Using western blot analysis, we further verified that NLRP3 was expressed in cardiac tissues in accordance with the previously mentioned immunohistochemistry findings (Fig. 7I, J).Furthermore, the outcomes of two additional proteins associated with pyroptosis, specifically caspase 1 and GSDMD-N, exhibited a substantial concurrence with the previously documented observations (Fig. 7E–H, K–M and L–N). The aforementioned findings align with the outcomes of in vitro cellular investigations. The findings suggest that suppressing the function of PLD2 in SICM mice could potentially attenuate cardiac tissue injury through modulation of the NLRP3/caspase-1/GSDMD-mediated pyroptosis pathway.

## Discussion

**Fig. 8:**
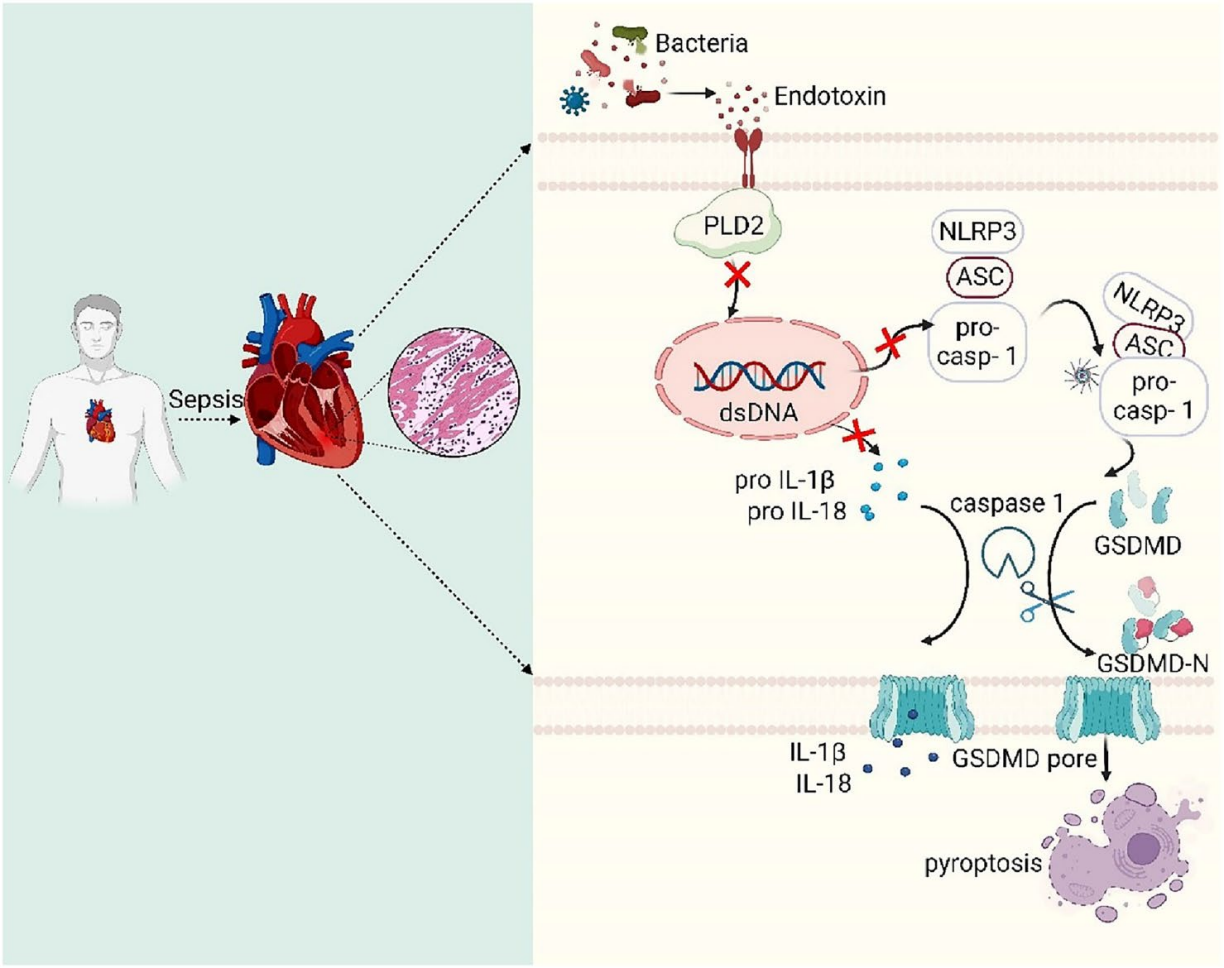
Mechanistic map of PLD2 deletion protects against SICM by suppressing pyroptosis via the NLRP3/caspase 1/ GSDMD pathway

SICM is a common complication in patients with sepsis that substantially increases mortality. An important pathophysiological feature of SICM is myocardial damage induced by the excessively inflammatory response of cardiomyocytes. In this study, myocardial damage and inflammatory responses were evident in mice with SICM, and cardiomyocyte PLD2 expression was significantly elevated. PLD2 deletion attenuates the inflammatory response of cardiomyocyte and ameliorates myocardial pathological changes and cardiomyocyte pyroptosis. By contrast, PLD2 overexpression exacerbates myocardial damage and leads to inflammation. Finally, PLD2 knockdown improved the survival of SICM mice. Our study’s findings indicated that PLD2 deletion alleviated the myocardial inflammatory response and cardiomyocyte damage involving NLRP3/caspase 1/GSDMD-mediated cardiomyocyte pyroptosis and improved the prognosis of the SICM model (Fig. 8).

PLD2 is expressed in cardiomyocytes and contributes to the development of several cardiac disorders by regulating inflammatory responses [14]. However, the involvement of PLD2 in the pathogenesis of SICM remains unknown. Gene microarray analysis of whole blood samples collected from individuals with sepsis revealed a notable positive association between the expression level of PLD2 and the rate of mortality observed in these patients [28]. Our previous study also demonstrated that PLD2 knockdown improved survival in mice with sepsis [17]. SICM is associated with a worse prognosis in sepsis, and PLD2 may decrease survival in patients with sepsis by exacerbating myocardial injury. In this study, the SICM mouse model was induced by CLP [26, 27]. Changes in cardiac histology, inflammatory factor levels, cardiac function, and indicators of myocardial damage were used to measure model construction success. The most serious cardiac involvement occurred 48 h after CLP stimulation. Western blotting demonstrated that the expression of PLD2 was upregulated in the cardiac tissues of SICM mice. These findings suggest that PLD2 is possibly involved in the pathophysiology of SICM.

We revealed an important function in the pathogenesis of SICM by altering the expression of the PLD2 gene. First, we generated PLD2 gene-specific knockout mice. The findings of this study demonstrated that mice with a deletion of the PLD2 gene have enhanced survival rates after CLP. We discovered that markers of myocardial injury, such as cTNI and LDH, were reduced in PLD2-knockdown mice. Cardiac ultrasonography revealed enhanced cardiac function, indicating that PLD2 deletion improved myocardial damage and cardiac function.

The inflammatory response is crucial in the pathogenesis of SICM [29–31]. Given the strong association between PLD2 and the regulation of inflammation, our study aimed to investigate the involvement of PLD2 in the inflammatory response of cardiomyocytes. Our study revealed a notable decrease in the production of inflammatory factors in shRNA-PLD2 cardiomyocytes following LPS stimulation. Additionally, the levels of inflammatory factors were lower in the cardiac tissues of PLD2-knockout SICM mice. These findings suggest that PLD2 has an essential role in alleviating the inflammatory response in the heart, thereby helping reduce myocardial damage.

Excessive bursts of inflammation can cause cellular damage via numerous mechanisms, including pyroptosis, a key mechanism of cellular destruction [21, 22, 32]. Pyroptosis can also produce inflammation. Pyroptosis is a type of programmed cell death mediated by GSDMD [24]. Activation of NLRP3 inflammasomes triggers pyroptosis in cardiomyocytes, leading to the progression of cardiac dysfunction and ultimately reducing the ejection fraction [33]. Pyroptosis is strongly correlated with cardiovascular ailments such as atherosclerosis, myocardial infarction, ischemia–reperfusion injury, and heart failure [34, 35]. Using scanning electron microscopy, we first examined the ultramicroscopic findings of cardiomyocytes and found that their structural integrity was compromised after stimulation with LPS. In particular, the cell membrane exhibited depressions and openings of various sizes, and the abundance of extracellular vesicles was notably diminished. Furthermore, NLRP3 expression was significantly elevated in the cardiac tissues of mice with SICM, which is consistent with the results of previous studies [25, 36]. These changes support the occurrence of cardiomyocyte pyroptosis in SICM. The inhibition of NLRP3 improves cardiac function in septic mice, thus implying that interference with NLRP3 inflammasome production may cure septic cardiomyopathy.

Whether PLD2 knockdown protects cardiac function in mice with SICM by inhibiting cardiomyocyte pyroptosis remains unclear. The expression of NLRP3 was reduced in PLD2-knockout mice. In vitro experiments revealed that downregulation of PLD2 reduced the expression of NLRP3, whereas the overexpression of PLD2 increased the expression of NLRP3. This finding strongly indicated that PLD2 has a regulatory role in cardiomyocyte pyroptosis. We examined the pathways involved in pyroptosis. The results obtained with western blotting and immunofluorescence analyses demonstrated an upregulation in the expression of pyroptosis-associated proteins—namely, NLRP3, caspase 1, and GSDMD—in H9C2 cardiomyocytes after stimulation with LPS. By contrast, the expression of pyroptosis-related proteins was decreased after PLD2 knockdown. Given the cellular biological processes associated with PLD2, the finding that specific knockout PLD2 animals have normal survival and physiological functions is noteworthy. The results of our study imply that PLD2 inhibitors as pharmaceutical interventions may exhibit therapeutic efficacy while presenting negligible health risks.

This study has some limitations. Septic cardiomyopathy is a reversible form of cardiac dysfunction. To determine the initial role of PLD2, we observed changes in PLD2 levels during the acute phase of the disease in a more severe model. Longer and more dynamic observations will help better understanding the role of PLD2 in this disease. Second, cardiac-specific knockout was not performed because of the common occurrence of multisystem involvement in septic cardiomyopathy. Given the systemic nature of the knockout, the possible extension of its effects to other interconnected systems is plausible. Third, we did not investigate the downstream targets of PLD2, such as phosphatidic acid, to identify specific signaling pathways.

In conclusion, PLD2 deletion is involved in SICM pathogenesis and is associated with the inhibition of the myocardial inflammatory response and pyroptosis through the regulation of the NLRP3/caspase 1/GSDMD pathway. This study provides a unique theoretical framework for future therapies and concepts related to the treatment of septic cardiomyopathy.

### Supplementary Information

Below is the link to the electronic supplementary material.

Supplementary file1 (DOCX 1517 KB)

Supplementary file2 (DOCX 18 KB)

## Data Availability

The datasets that support the conclusions of this article are included in the article.
