# Supplementary material for: PLD2 deletion ameliorates sepsis-induced cardiomyopathy by suppressing cardiomyocyte pyroptosis via the NLRP3/caspase 1/GSDMD pathway
Source: Inflamm Res. 2024 Apr 17;73(6):1033–46. doi: 10.1007/s00011-024-01881-w (PMC11106193; doi:10.1007/s00011-024-01881-w)

**Electronic supplemental material**

**PLD2 deletion ameliorates sepsis-induced cardiomyopathy by suppressing cardiomyocyte pyroptosis via the NLRP3/ caspase 1/GSDMD pathway**

**Contents**

Supplemental Figure 1

Supplemental Figure 2

Supplemental Figure 3

Supplemental Figure 4

Supplemental Figure 5

Supplemental Figure 6

**Supplemental Fig. 1** Original western blot for three repeats (Fig. 1 K)

**
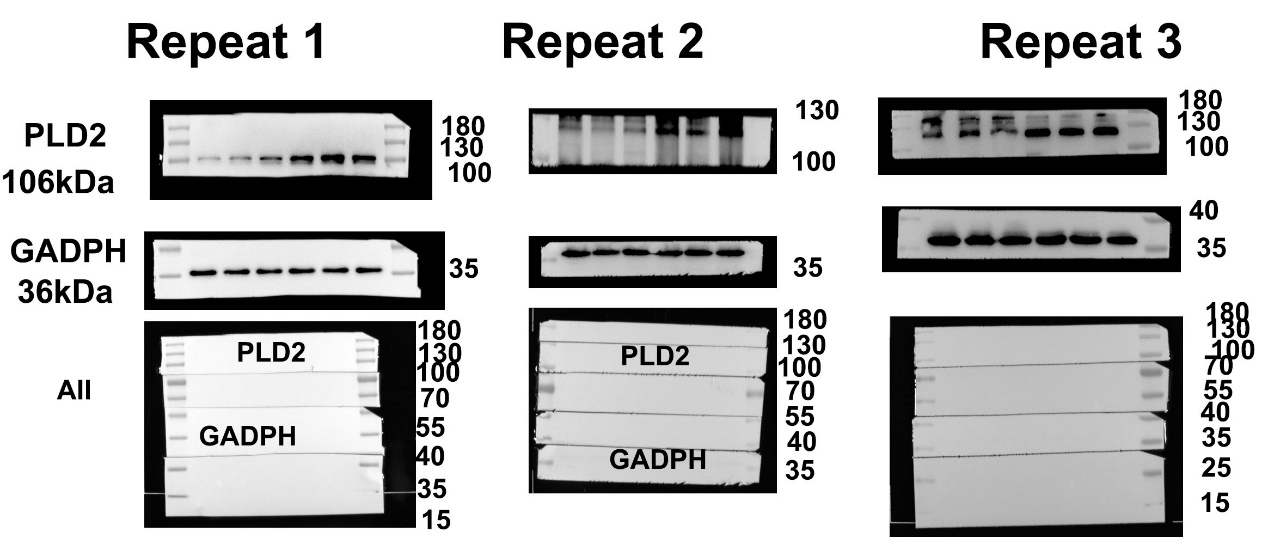
**

**Supplemental Fig. 2** Original western blot for three repeats (Fig. 2 A)

**
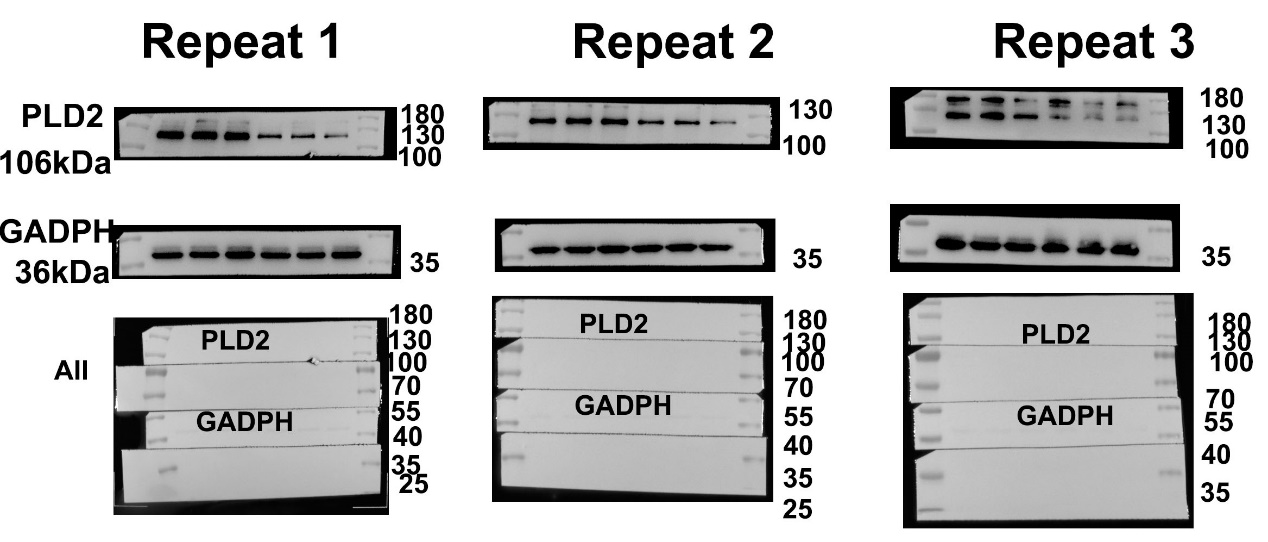
**

**Supplemental Fig. 3** Original western blot for three repeats (Fig. 4 B, E and H)


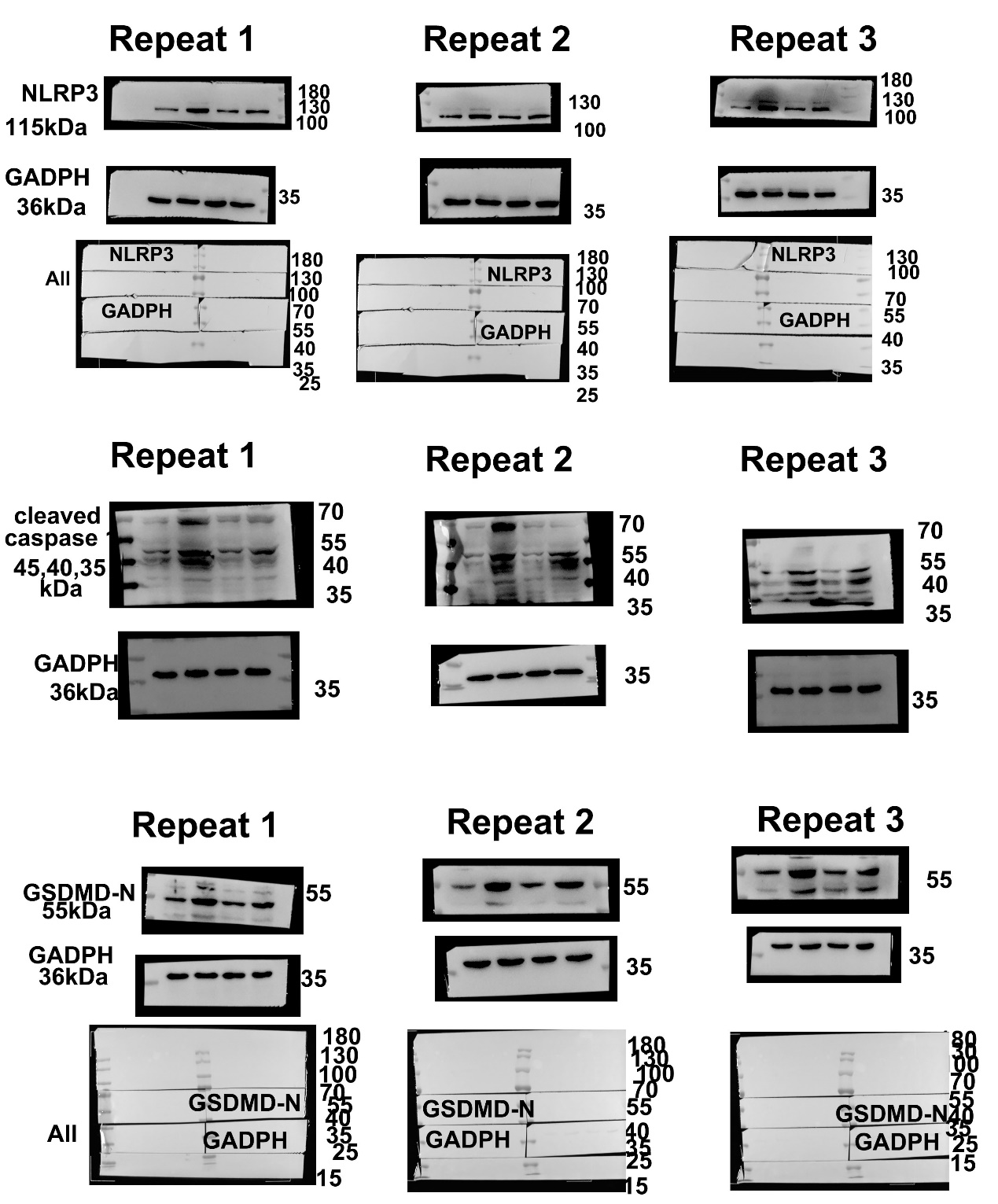


**Supplemental Fig. 4** Original western blot for three repeats (Fig. 5 A and B)

**
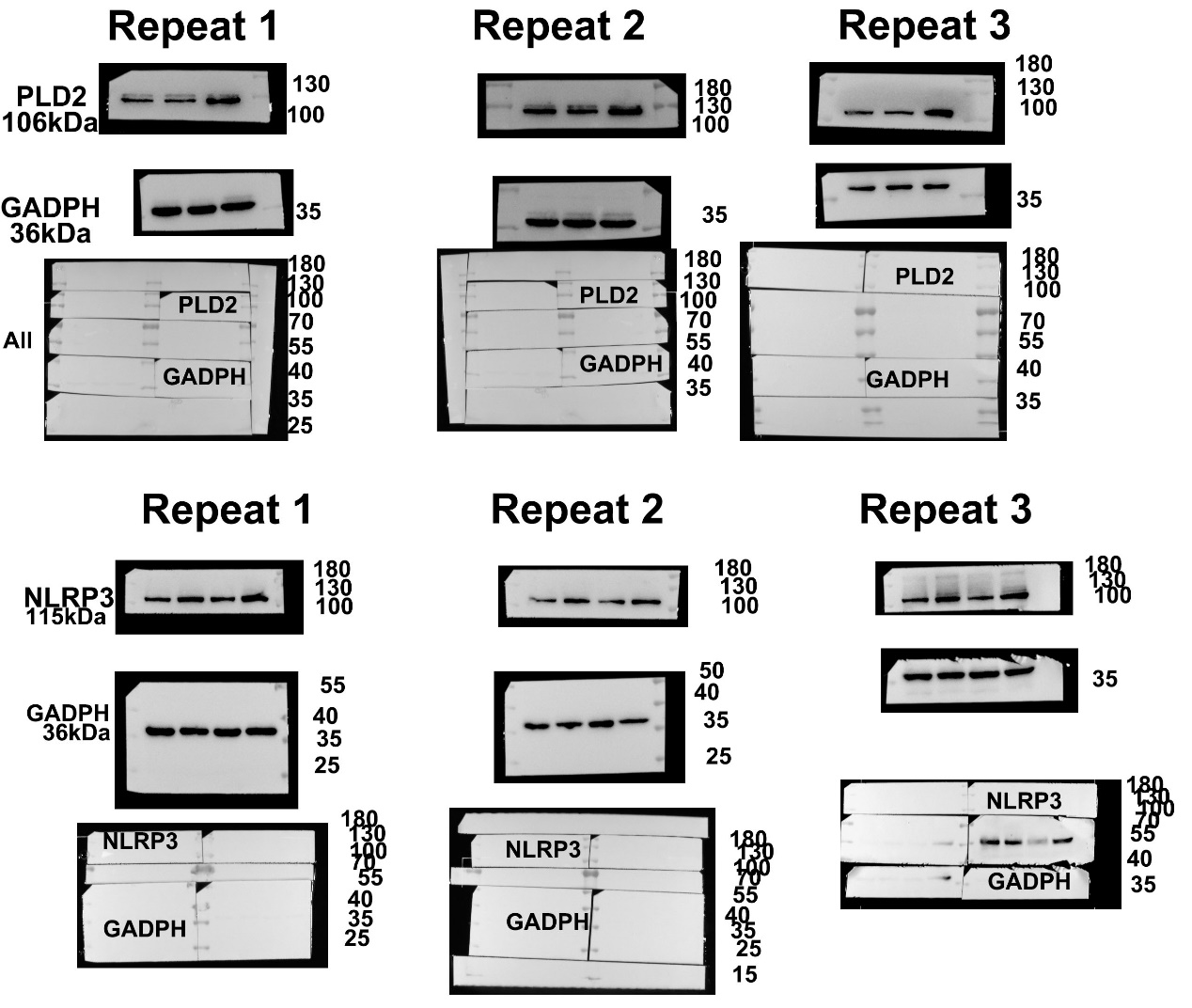
**

**Supplemental Fig. 5** Original western blot for three repeats (Fig. 6 A)

**
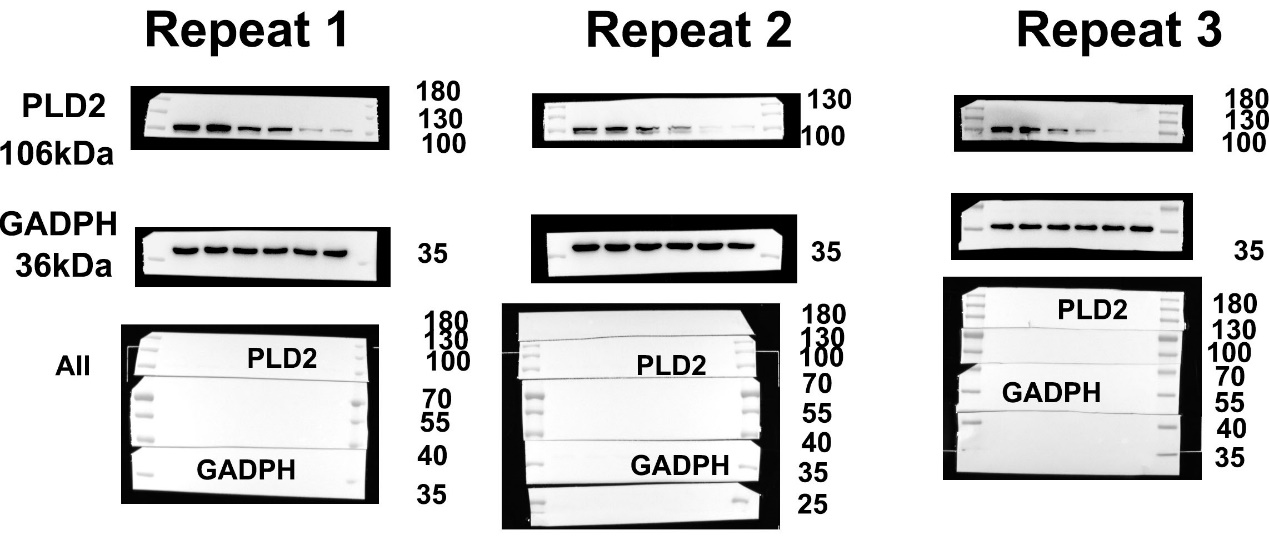
**

**Supplemental Fig. 6** Original western blot for three repeats (Fig. 7 I, K and M)


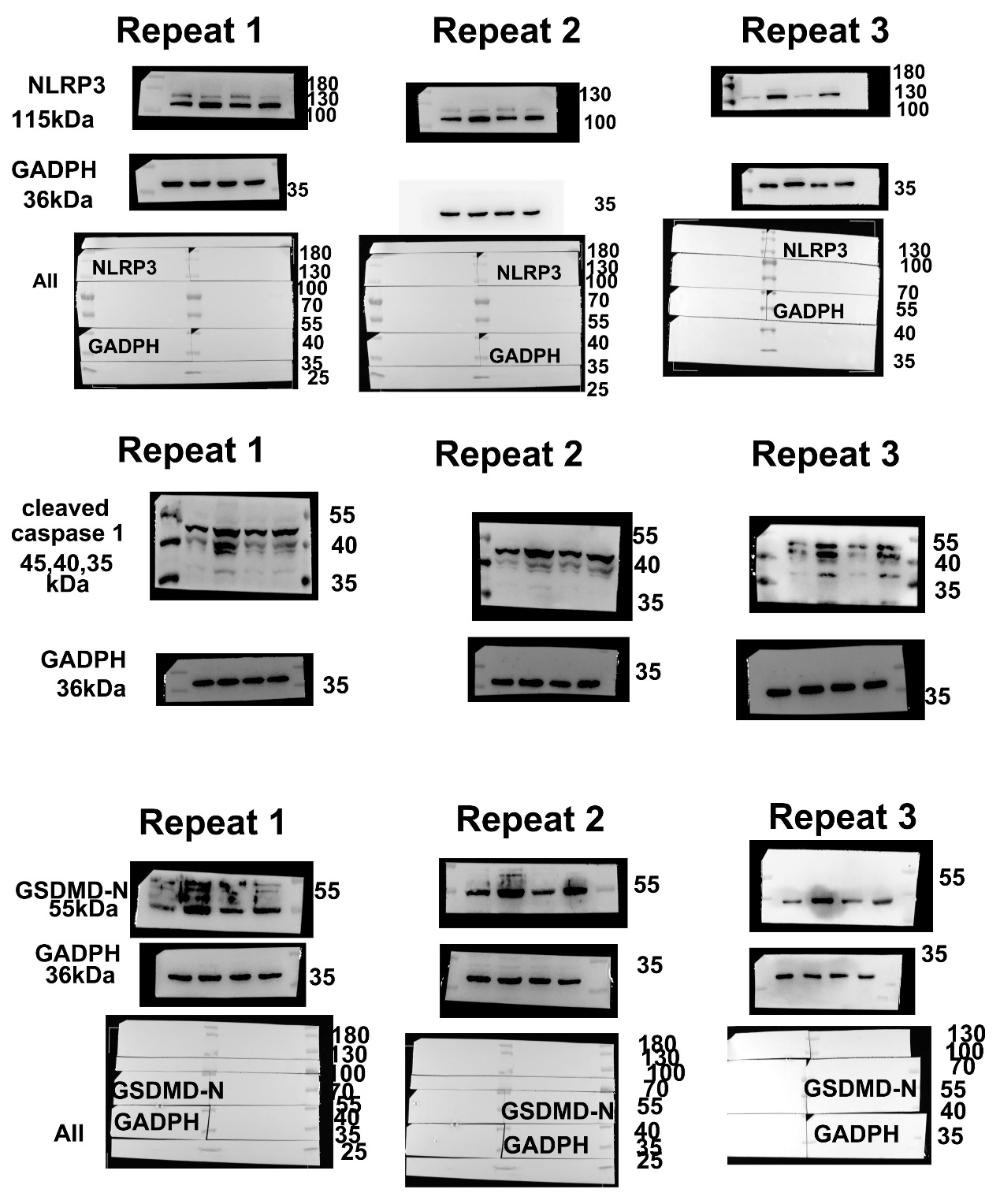

Supplement: Supplementary file 1 — Supplementary file1 (DOCX 1517 KB) [file 11_2024_1881_MOESM1_ESM.docx]
