# Supplementary material for: PLD2 deletion ameliorates sepsis-induced cardiomyopathy by suppressing cardiomyocyte pyroptosis via the NLRP3/caspase 1/GSDMD pathway
Source: Inflamm Res. 2024 Apr 17;73(6):1033–46. doi: 10.1007/s00011-024-01881-w (PMC11106193; doi:10.1007/s00011-024-01881-w)
Supplement: Supplementary file 2 — Supplementary file2 (DOCX 18 KB) [file 11_2024_1881_MOESM2_ESM.docx]

**Electronic supplemental material**

**PLD2 deletion ameliorates sepsis-induced cardiomyopathy by suppressing cardiomyocyte pyroptosis via the NLRP3/ caspase 1/GSDMD pathway**

**Contents**

Supplemental Table 1

Supplemental Table 2

**Supplemental Table 1.** Echocardiographic parameters of cardiac function in mice following CLP.

| Echocardiographic  parameter | Name of experimental groups | | |
| --- | --- | --- | --- |
|  | Control | CLP-24 h | CLP-48 h |
| LVEF（%） | 65.12±2.48 | 50.56±3.04** | 33.49±3.55** |
| LVFS（%） | 34.49±2.44 | 24.86±1.84** | 15.31±1.76** |
| LVEDV（μL） | 55.29±6.77 | 39.35±2.70* | 38.32±7.32* |
| LVESV（μL） | 19.71±3.52 | 19.47±2.03 | 25.67±5.85 |
| LVIDd（mm） | 3.62±0.18 | 3.14±0.09* | 3.10±0.24* |
| LVIDs（mm） | 2.37±0.16 | 2.36±0.10 | 2.63±0.24 |
| CO ( ml/min) | 12.91±1.54 | 7.57±0.43** | 5.75±0.77** |
| HR（bpm） | 363.50±22.94 | 381.83±16 | 455.00±18.73** |

Left ventricular ejection fraction (LVEF), left ventricular shortening fraction (LVFS), left ventricular volumes at end diastole (LVEDV) and systole (LVESV), left ventricular internal diameter at end-diastole (LVIDd) and left ventricular internal diameter at end-systole (LVIDs),

cardiac output (CO), heart rate (HR). Data were expressed as the means ± SD, n=6 (each group).

*p<0.05, **p<0.001 vs. control.

**Supplemental Table 2.** Effect of PLD2 deletion on CLP-induced SICM mice changes in echocardiographic measurements of cardiac function parameters.

| Echocardiographic  parameter | Name of experimental groups | | | |
| --- | --- | --- | --- | --- |
|  | PLD2^+/+^ | PLD2^+/+^+CLP-48 h | PLD2^-/-^ | PLD2^-/-^+CLP-48 h |
| LVEF（%） | 65.12±2.48 | 33.49±3.55** | 63.82±4.34 | 50.12±4.94^##^ |
| LVFS（%） | 34.49±2.44 | 15.31±1.76** | 34.04±3.10 | 24.64±2.93^##^ |
| LVEDV（μL） | 55.29±6.77 | 38.32±7.32* | 54.07±0.09 | 39.81±5.45 |
| LVESV（μL） | 19.71±3.52 | 25.67±5.85 | 19.80±5.37 | 19.92±4.25 |
| LVIDd（mm） | 3.62±0.18 | 3.10±0.24* | 3.58±0.25 | 3.15±0.19 |
| LVIDs（mm） | 2.37±0.16 | 2.63±0.24 | 2.51±0.41 | 2.37±0.20 |
| CO ( ml/min) | 12.91±1.54 | 5.75±0.77** | 12.42±1.58 | 7.69±0.66^##^ |
| HR（bpm） | 363.50±22.94 | 455.00±18.73** | 363.83±21.81 | 392.50±33.79^#^ |

Left ventricular ejection fraction (LVEF), left ventricular shortening fraction (LVFS), left ventricular volumes at end diastole (LVEDV) and systole (LVESV), left ventricular internal diameter at end-diastole (LVIDd) and left ventricular internal diameter at end-systole (LVIDs),

cardiac output (CO), heart rate (HR). Data were expressed as the means ± SD, n=6 (each group).

*p<0.05, **p<0.001 vs. control. ^#^ p<0.05, ^##^p<0.001 vs. PLD2^+/+^+CLP-48 h.
